# Access of Hydrogen-Radicals to the Peptide-Backbone as a Measure for Estimating the Flexibility of Proteins Using Matrix-Assisted Laser Desorption/Ionization Mass Spectrometry

**DOI:** 10.3390/ijms15058428

**Published:** 2014-05-13

**Authors:** Mitsuo Takayama, Keishiro Nagoshi, Ryunosuke Iimuro, Kazuma Inatomi

**Affiliations:** Graduate School of Nanobioscience, Mass Spectrometry Laboratory, Yokohama City University, 22-2 Seto, Kanazawa-ku, Yokohama 236-0027, Japan; E-Mails: knagoshi@yokohama-cu.ac.jp (K.N.); n135202e@yokohama-cu.ac.jp (R.I.); i100078g@yokohama-cu.ac.jp (K.I.)

**Keywords:** proteins, hydrogen-radical, flexibility, MALDI MS (matrix-assisted laser desorption/ionization mass spectrometry), in-source decay

## Abstract

A factor for estimating the flexibility of proteins is described that uses a cleavage method of “in-source decay (ISD)” coupled with matrix-assisted laser desorption/ionization mass spectrometry (MALDI MS). The MALDI-ISD spectra of bovine serum albumin (BSA), myoglobin and thioredoxin show discontinuous intense ion peaks originating from one-side preferential cleavage at the N-Cα bond of Xxx-Asp, Xxx-Asn, Xxx-Cys and Gly-Xxx residues. Consistent with these observations, Asp, Asn and Gly residues are also identified by other flexibility measures such as B-factor, turn preference, protection and fluorescence decay factors, while Asp, Asn, Cys and Gly residues are identified by turn preference factor based on X-ray crystallography. The results suggest that protein molecules embedded in/on MALDI matrix crystals partly maintain α-helix and that the reason some of the residues are more susceptible to ISD (Asp, Asn, Cys and Gly) and others less so (Ile and Val) is because of accessibility of the peptide backbone to hydrogen-radicals from matrix molecules. The hydrogen-radical accessibility in MALDI-ISD could therefore be adopted as a factor for measuring protein flexibility.

## 1. Introduction

Mass spectrometry (MS) is an indispensable tool for analyzing biological molecules such as protein, nucleic acid, saccharides, hormones, lipid and neurotransmitters in living organisms. In particular, matrix-assisted laser desorption/ionization (MALDI) [1,2] and electrospray ionization (ESI) [3,4] have become widely recognized as powerful soft ionization methods for identifying the expressed proteome. Although both MALDI MS and ESI MS have outstanding abilities that allow identification of proteins due to their rapidity and high sensitivity, it is more difficult to glean the kind of information about secondary and tertiary structures that can be determined by X-ray crystallography and nuclear magnetic resonance (NMR) spectroscopy. However, recent reports about the relationships between MS and NMR data of cytochrome *c* suggest that MALDI [5,6] and ESI [7] might be useful for estimating flexible regions of proteins.

One of the recent interests in protein science is the concept of flexibility by means of the motion of backbone and/or side chains. It is believed that the flexibility of protein molecules relates to several functions such as post-translational modifications and drug interactions. The concept of intrinsically disordered proteins has been recognized as key in clarifying functions with respect to intermolecular interactions with other proteins, nucleic acids and drug chemicals [8]. The flexibility of protein molecules is estimated by several measures such as the B-factor [9,10], the turn preference factor [11,12], the protection factor [13] and the fluorescence decay factor [14]. The B-factor and turn preference factor are based on X-ray crystallography in the crystal phase, while the protection factor is based on the hydrogen/deuterium exchange (HDX) reaction in NMR and the fluorescence decay factor can be obtained from protein molecules in solution. The B-factor which relates to side chain mobility suggests that Asp, Asn, Gly, Pro, Lys, Glu, Gln and Ser residues have a more flexible nature than other residues [15]. The turn preference factor indicates that Asp, Asn, Gly, Pro, Cys and Ser residues do not favor intra-molecular hydrogen bonded secondary structures such as α-helix and β-sheet [11,12]. The flexible residues estimated by the protection factor from NMR-HDX experiments of ubiquitin and cytochrome *c* have been reported to be Asp, Asn, Gly, Lys, Thr, Ile and Met [13,16]. The fluorescence decay factor shows that Asp, Asn, Gly, Ser and Ala are more flexible than the other residues [14]. Although the residues estimated are intrinsic to that method used, it is interesting that Asp, Asn and Gly residues are common to all the methods, independent of the principle and experimental conditions such as crystal and solution phase.

**Scheme 1.**
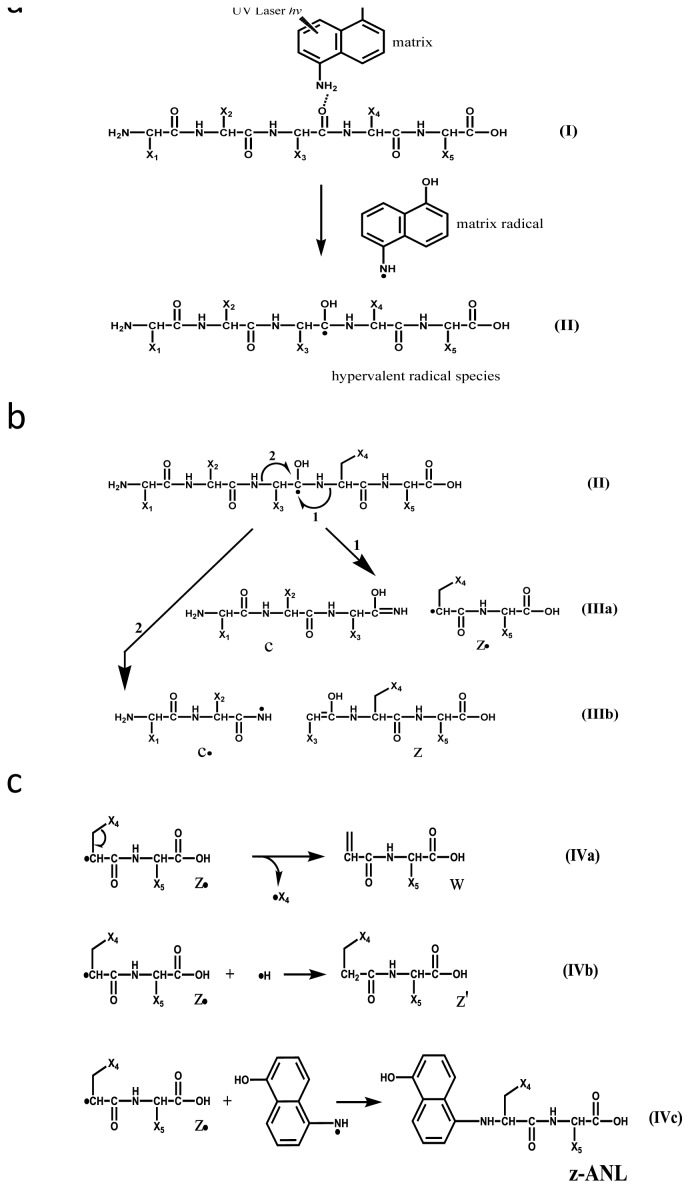
(**a**) Interaction between matrix active-hydrogens and carbonyl oxygens of the peptide backbone, hydrogen transfer from matrix to the backbone and the formation of hypervalent radical species of protein. (**b**) Pathways of N-Cα Bond Cleavage of the Backbone to generate Fragment c/z• and c•/z Pairs and the Nomenclature of ISD (in-source decay) Fragments and (**c**) Formation of ISD Fragments w, z′ and z-ANL.

We recently reported that Asp, Asn, Gly and Cys residues in equine cytochrome *c* susceptible to in-source decay (ISD) coupled with MALDI-ISD were partly consistent with them being in flexible regions as estimated by the protection factor in NMR-HDX experiments [6,17]. This suggested that MALDI-ISD could be used as a method for estimating the flexibility of proteins. The MALDI-ISD experiments were performed by irradiating the surface of the matrix crystals, in which were embedded analyte protein molecules, with ultraviolet (UV) pulsed laser photons under vacuum conditions. The irradiation of the crystal phase with UV-laser photons results in ablation (to generate a dense-gas plume), proton transfer (to generate analyte ions), and hydrogen-radical transfer (to produce the ISD reaction). ISD is a radical-initiated cleavage at the N-Cα bond of the peptide backbone, and the processes can be divided into two steps: (a) the formation of hypervalent radical species of protein molecules (Scheme 1a) and (b) prompt cleavage (within several tens of nanoseconds in the ion source) to generate ISD fragments c, z′, z-matrix and w (Scheme 1b,c) [18,19]. The formation of the ISD fragments can be rationalized by so-called “Takayama’s model” [19]. According to the model, the hydrogen-radicals are generated from active-hydrogens (−OH and −NH_2_) of the matrix molecules excited with UV photons [20–22]. However, reasons why the specific residues of Asp, Asn, Gly and Cys are more susceptible to ISD than other residues and why the one-side preferential cleavage at the N-Cα bond of the residues (Xxx-Asp, Xxx-Asn, Gly-Xxx and Xxx-Cys) occurs have not yet been rationalized, in spite of a related previous report [23]. It is of particular interest to consider the influence of secondary structures of proteins on the ISD fragment abundance, although it is difficult to obtain any evidence for the presence of α-helix and β-sheet for a given protein in the MALDI matrix crystal state and/or MS gas-phase conditions.

Here we describe relationships between discontinuous intense peaks in the ISD spectra of analyte proteins and secondary structures based on the X-ray and NMR data. The proteins used are bovine serum albumin (BSA), equine myoglobin and human thioredoxin, for which there are primary, secondary and tertiary structures as determined by X-ray crystallography and NMR spectroscopy, and are recorded in the protein data bank (PDB). The ISD spectra showing fragment peaks of c-, z′- and w-ions are discussed from definite secondary structures as judged by means of X-ray and NMR data. The reactions of N-Cα bond cleavage and one-side preferential cleavage at the N-Cα bond of Xxx-Asp/Asn/Cys and Gly-Xxx are discussed on the basis of *ab initio* calculations. The results indicate that the one-side preferential N-Cα bond cleavage may be due to hydrogen bonding interactions between matrix active-hydrogen(s) and the carbonyl oxygens of the backbone of protein molecules embedded in/on the matrix crystal. Furthermore, it maybe that the protein molecules embedded in/on matrix crystals may partially maintain their helix structure, and that the carbonyl oxygens of helix-free backbone regions of the protein molecules embedded in/on matrix crystals are exposed to the matrix molecules and are easily accessed by matrix active-hydrogens. It is demonstrated that hydrogen-radical accessibility to the peptide-backbone is a factor for estimating the flexibility of protein and is compatible with the turn preferential factor for the flexible amino acid residues Asp, Asn, Gly and Cys.

## 2. Results and Discussion

### 2.1. Preferential Binding of Hydrogen-Radicals Determining One-Side Preferential Cleavage at N-Cα Bond of Peptide Backbone

**Scheme 2.**
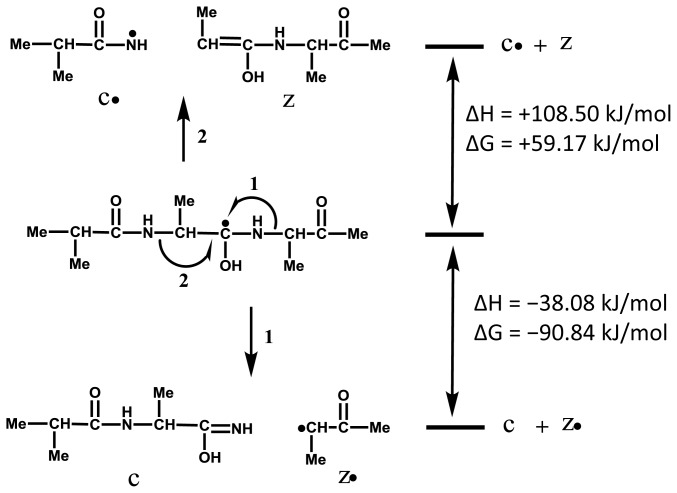
Thermochemical Values ΔG (kJ/mol) and ΔH (kJ/mol) for the Formation of ISD Fragments c/z• and c•/z pairs obtained from *ab initio* Calculations.

It is important to divide the overall ISD process into two parts, (a) the formation of hypervalent radical species II (Scheme 1a), and (b) the generation of ISD fragments (Scheme 1b,c). For the fragment formation in Scheme 1b, it needs be confirmed which reaction is energetically favorable for the formation of either the c/z•-pair (reaction 1) or the c•/z-pair (reaction 2) in Scheme 1b (II). In order to avoid any intra-molecular hydrogen bonding in the analyte, here we use a methylated model tri-peptide (Me-Ala-Ala-Ala-Me) for *ab initio* calculations. The calculations were performed for a radical species by which radical site was located on the carbonyl carbon at Ala2 residue (Scheme 2). The calculation results indicate that reaction 1 is overwhelmingly favorable over reaction 2, as shown in Scheme 2. Further, the results support a fact that the ISD spectra never give c-ANL fragment ions [6,19] originated from a radical fragment c• (IIIb in Scheme 1b), while z-ANL fragment ions originated from a radical fragment z• can be observed in the ISD spectra [6,19]. We can therefore employ reaction 1 for the formation and nomenclature of ISD fragments (Scheme 1b).

**Scheme 3.**
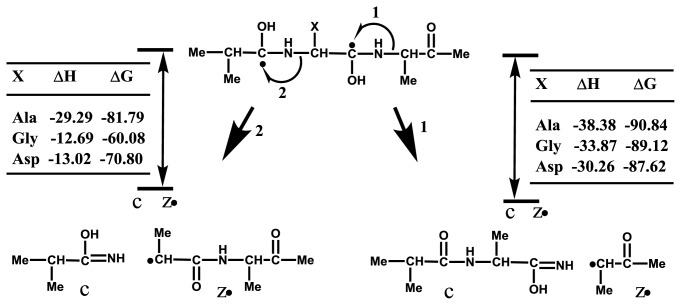
Thermochemical Values ΔH (kJ/mol) and ΔG (kJ/mol) for the Formation of c/z•. Pairs Originating from Cleavages at the N-Cα bond of Both Sides of n-th Residues Xxx-X and X-Xxx.

To estimate the one-side preferential cleavage at the N-Cα bond of n-th residue Xxx-Xn or Xn-Xxx, *ab initio* calculations were performed for the model peptide Me-Ala-X-Ala-Me (X = Ala, Gly, Asp). Both calculated enthalpy ΔH and Gibbs energy ΔG showed that *C*-terminal side cleavage (Xn-Xxx) is more favorable than the *N*-terminal side (Xxx-Xn), as shown in Scheme 3. The result supports the preferential cleavage at the N-Cα bond of Gly-Xxx, but does not explain the even cleavage of Xxx-Ala and Ala-Xxx and the preferential cleavage at Xxx-Asp. On the basis of the calculations, it is suggested that the one-side preferential cleavage is not governed by the energetics of reactions 1 and 2 in Scheme 3. It seems reasonable to consider that the one-side preferential cleavage is attributable to preferential binding of hydrogen-radicals to the *N*-terminal side carbonyl oxygens (CO-NH-Asp/Asn/Cys) and *C*-terminal side carbonyl oxygens (Gly-CO). This indicates that accessibility of hydrogen-radicals to the backbone carbonyl oxygens of analyte proteins is essential for the preferential cleavage leading to the observation of discontinuous intense ISD fragment ions. It is of importance to note that the backbone regions easily accessible by hydrogen-radicals or matrix molecules have to be lying in flexible secondary structures.

### 2.2. Discontinuous Intense ISD (In-Source Decay) Ion Peaks Reflect Helix-Free Regions of Protein

**Figure 1.**
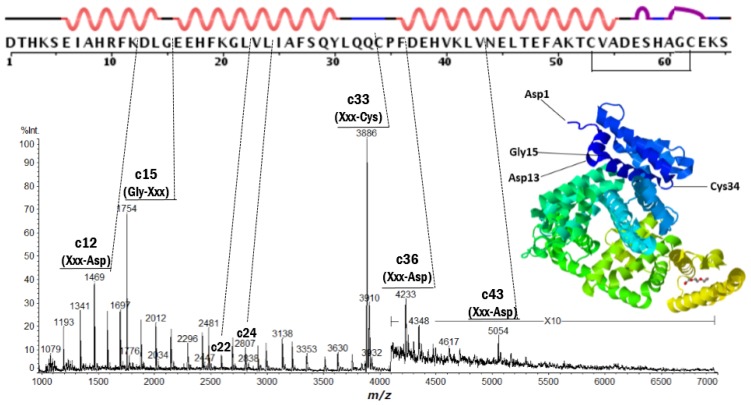
Positive ion matrix-assisted laser desorption/ionization in-source decay (MALDI-ISD) spectrum of bovine serum albumin (*M*r 66430.3) obtained with 5-amino-1-naphthol matrix. The inset represents tertiary structure obtained by X-ray crystallography (PDB: 4F5S). The upper line represents primary and secondary structure (wave: α-helix, convex: turn, straight: bend or empty).

**Figure 2.**
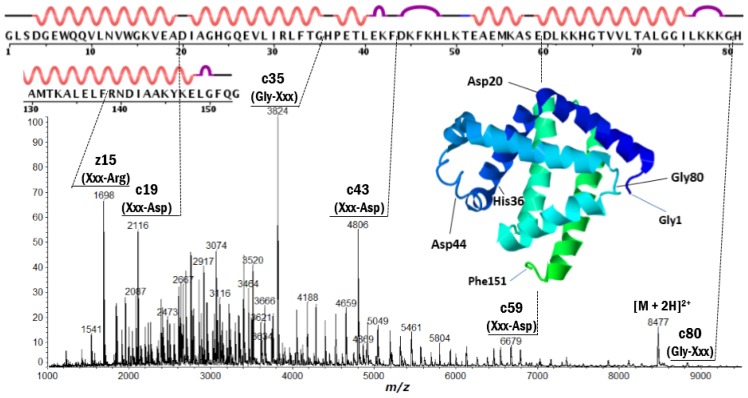
Positive ion MALDI-ISD spectrum of equine apo-myoglobin (*M*r 16951.4) obtained with 5-amino-1-naphthol matrix. The inset represents tertiary structure obtained by X-ray crystallography (PDB: 2FRF). The upper line represents primary and secondary structure (wave: α-helix, convex: turn, straight: bend or empty). Adapted from [18] with permissions from Springer, copyright 2014.

Positive ion MALDI-ISD spectra of bovine serum albumin (BSA) and equine apo-myoglobin obtained with 5-amino-1-naphthol (5,1-ANL) as a matrix (which is a suitable matrix for MALDI-ISD experiments with protein [23,24]) are shown in Figures 1 and 2, respectively. The ISD spectra showed remarkably discontinuous intense peaks assigned as the fragments c-, z′- and w-ions (Table 1). The ionization (protonation and deprotonation) of the ISD fragments takes place independent of the ISD processes [25], and the ISD fragment ions are usually observed as singly-charged ions, *i.e.*, c-ion = [c + H]^+^ and [c − H]^−^, z′-ion = [z′ + H]^+^ and [z′ − H]^−^, z-matrix ion = [z-matrix + H]^+^ and [z-matrix − H]^−^, and w-ion = [w + H]^+^ and [w − H]^−^. The primary structures and ISD fragment ions (intensity) observed for the analyte proteins used here are presented in Supplementary Information 1 and 2, respectively.

**Figure 3.**
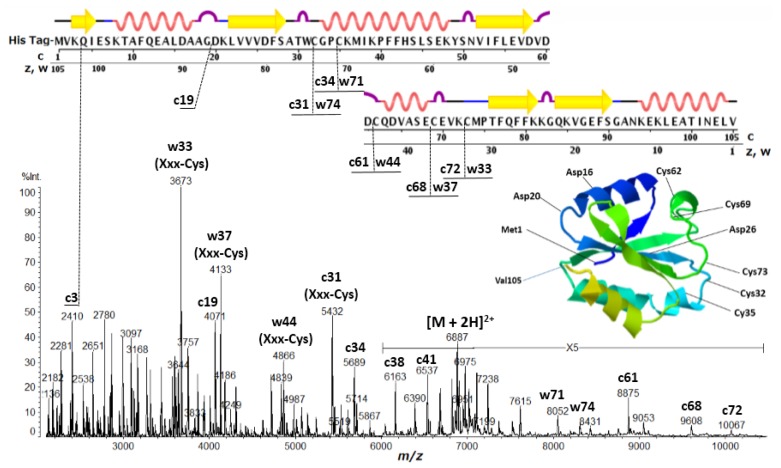
Positive ion MALDI-ISD spectrum of human thioredoxin with HisTag (*M*r 13,769.6) obtained with 5-amino-1-naphthol matrix. The inset represents tertiary structure obtained by X-ray crystallography (PDB (protein data bank): 1AUC). The upper line represents primary and secondary structure (wave: α-helix, arrow: β-strand, convex: turn, straight: bend or empty).

It is important to note here that the peak abundance of the ISD fragment ions is dependent upon two factors: (A) the preferential cleavage at the N-Cα bond of the peptide backbone; and (B) the presence of basic residues (Arg, His, Lys and *N*-terminus) or acidic residues (Glu, Asp and *C*-terminus) to give positive or negative ions. The factor B refers to the ionization efficiency *I* (ions/molecules) for the formation of analyte and fragment ions. It is well known that the presence of basic amino acid residues (Arg, Lys, His) and the *N*-terminus generally increases the abundance of the analyte and fragment ions [26,27]. A very intense peak corresponding to *C*-terminal side ISD ion z′15 in Figure 2 can be explained by the appearance of Arg15 residue from *C*-terminus. In the ISD spectrum of thioredoxin (Figure 3), the *N*-terminal side intense c3 ion corresponding to the HisTag-MVK (Met-Val-Lys) may be due to the presence of the Lys3 residue. However, other discontinuous intense peaks observed in Figures 1–3 cannot be explained by the charge sites (factor B).

With respect to factor A, the access of active-hydrogens of matrix molecules to carbonyl oxygens of the backbone is influenced by some of the physical characteristics of a given protein, such as intra-molecular hydrogen-bonded secondary structures (α-helix and β-sheet) and bulky side chains resulting in steric hindrance that inhibits the access of the hydrogen-radicals. It may be expected that the presence of α-helix and β-sheet and the bulky side chains may inhibit any preliminary hydrogen-bonding between matrix active-hydrogens and backbone carbonyl oxygens. Although it is difficult to estimate the presence of α-helix and β-sheet in protein molecules embedded in/on the MALDI matrix crystals, the observation of remarkably discontinuous intense peaks for c15 and c33 ions in the ISD spectrum of BSA (Figure 1) strongly suggests that the helix segments as determined by X-ray crystallography are maintained, even when BSA molecules are lying in the matrix crystal. However, the observation of a lot of c-ions (*m/z* 1000–5000) indicates that the helix segments are incompletely maintained in the matrix crystal phase. Furthermore, the ISD spectrum of myoglobin also showed discontinuous intense peaks of c19, c35, c43 and c80 originating from cleavage at the N-Cα bond of Xxx-Asp or Gly-Xxx residues lying in helix-free regions. Recently, we reported that positive ion collision-induced dissociation (CID) of c35 and c43 ions generated from MALDI-ISD resulted in a remarkable product ion y23 originating from the cleavage at the peptide bond between Asp20-Ile21 residues lying in a helix-free region between two long helical domains [18], while the negative ion CID did not produce any readable product ions. This suggested that gas-phase fragment ions c35 and c43 generated from the MALDI-ISD of myoglobin maintain α-helix structure. It should be noted here that the secondary structures determined by X-ray and NMR of proteins (myoglobin (PDB: 2FRF (X-ray), 1MYF (NMR)), cytochrome *c* (PDB: 1HRC (X-ray), 1AKK (NMR)) and thioredoxin (PDB: 1AUC (X-ray), 3TRX (NMR))) are similar to each other. This indicates that secondary structures of the proteins are energetically stable in both crystal and solution phases. From this, it may be deduced that protein molecules embedded in/on matrix crystals partly maintain their secondary structures as determined by X-ray and NMR. Regarding this, here we have to pay attention to the denaturation of protein molecules which might be caused through solution preparation and crystallization. The denaturation of protein molecules would occur via two processes such as unfolding of native tertiary structures and destroy of secondary structures made of intra-molecular hydrogen bonding. The excess amounts of solvent and matrix molecules in MALDI sample solutions might cause the denaturation via inter-molecular interactions. It should be noted however that relatively long α-helix structures made of multi-hydrogen bondings are stable by a cooperative effect even when acids were added [28]. So, it seems to be reasonable to assume that protein molecules embedded in/on MALDI matrix crystals partly maintain their secondary structures.

Thioredoxin contains five Cys residues which do not form disulfide bonds. The ISD spectrum showed discontinuous intense peaks of c3 (Lys-Xxx), c19 (Gly-Xxx), c25 (Xxx-Asp), c31 (Xxx-Cys), c34 (Xxx-Cys), c61 (Xxx-Cys), c68 (Xxx-Cys), c72 (Xxx-Cys), w33 (Xxx-Cys), w37 (Xxx-Cys), w44 (Xxx-Cys), w71 (Xxx-Cys) and w74 (Xxx-Cys). The most notable characteristic in the ISD of thioredoxin is cleavage at the N-Cα bond of Xxx-Cys residues to generate w-ions accompanied by the loss of the SH (thiol) group from the side chain (Scheme 1b). The Cys residues in thioredoxin lie in the helix- and sheet-free regions, with the exception of Cys35 (upper in Figure 3). The one-side preferential cleavage at the *N*-terminal side N-Cα bond of Cys residues (Xxx-Cys) is also observed in the ISD spectrum of BSA (Figure 1). This suggests that the *N*-terminal side of Cys (CO-NH-Cys) exposes its carbonyl oxygen to the matrix active-hydrogens more than that on the *C*-terminal side of Cys residues (Cys-CO). A further characteristic of the ISD of thioredoxin is the formation of a c/w-ion pair originating from N-Cα bond cleavage of Xxx-Cys residues, *i.e.*, c31/w74, c34/w71, c61/w44, c68/w37 and c72/w33. The sulfur atom has a capability of the binding of electron or unpaired electron, and the Cys residue tends to appear in turns and to disrupt α-helices. It may therefore be expected that Cys residues expose the adjacent backbone region to matrix molecules resulting in preliminary hydrogen-bonding between backbone carbonyl oxygens or the sulfur atom and matrix active-hydrogens in the MALDI matrix crystal embedded protein molecules.

### 2.3. Accessibility of Carbonyl Oxygens of the Backbone to Hydrogen-Radicals

For the one-side preferential N-Cα bond cleavage that leads to the formation of abundant c-, z′- and w-ions, it can be considered that the hydrogen radicals or matrix active-hydrogens interact with the *N*-terminal side carbonyl oxygens of Xxx-Asp/Asn/Cys and the *C*-terminal side carbonyl oxygens of Gly-Xxx residues. This indicates that those carbonyl oxygens are free from intra-molecular hydrogen bonded secondary structures such as α-helix and β-sheet. With respect to this, it is important to take into account the secondary structure preference of amino acids [11,12]. According to the report of Chou *et al*. [11], Asn, Gly, Pro, Asp, Ser and Cys residues are preferred in turn structure. In particular, Asn, Cys and Asp residues appear at the start of β-turn regions with high probability. It should be noted further that Gly, Cys, Ser, Pro and Tyr residues tend to destroy helix structure. The preferential cleavage of Xxx-Asp, Xxx-Asn, Xxx-Cys and Gly-Xxx residues as ascertained here is consistent with the criteria for turn preference of amino acids [11,12], with the exception of the Pro residue. The Pro residue gives incomplete cleavage in ISD, because of its ring structure. As expected, the turn regions lie on the surface of protein molecules, so that the turn preferred residues tend to expose their backbone and side-chains to the environment including matrix molecules in MALDI crystal phase. In other words, the turn preferred residues are highly accessible to the matrix molecules in MALDI crystal phase. Therefore, the one-side preferential N-Cα bond cleavage of Xxx-Asp/Asn/Cys and Gly-Xxx residues in the MALDI-ISD experiments here may be due to high accessibility of the carbonyl oxygens located in the Xxx regions to hydrogen-radicals or matrix active-hydrogens.

**Figure 4.**
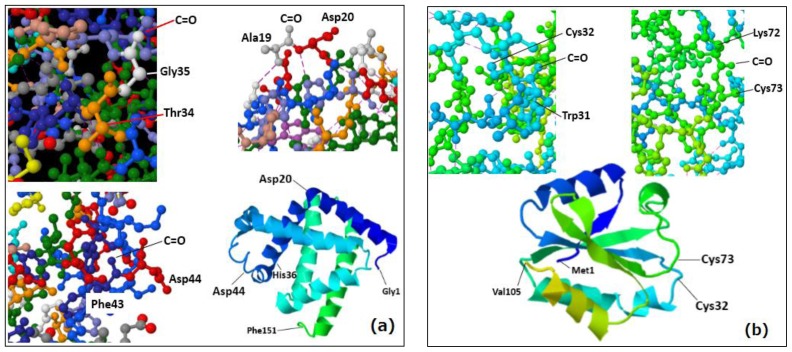
The sites of carbonyl groups at (**a**) Xxx-Asp20, Xxx-Asp44 and Gly35-Xxx residues obtained from the X-ray crystallography structure of myoglobin (PDB: 2FRF); (**b**) Xxx-Cys32 and Xxx-Cys73 residues of thioredoxin (PDB: 1AUC).

To help illustrate the point Figure 4 shows the sites of carbonyl groups at Xxx-Asp20, Xxx-Asp44 and Gly35-Xxx residues obtained from the X-ray data of myoglobin (PDB:2FRF). The carbonyl groups shown here have oxygen atoms exposed to the surroundings without intra-molecular hydrogen bonding. The carbonyl groups at Xxx-Cys32 and Xxx-Cys73 residues of thioredoxin (PDB: 1AUC) also have their oxygen atoms exposed to the surroundings (Figure 4b).

**Scheme 4.**
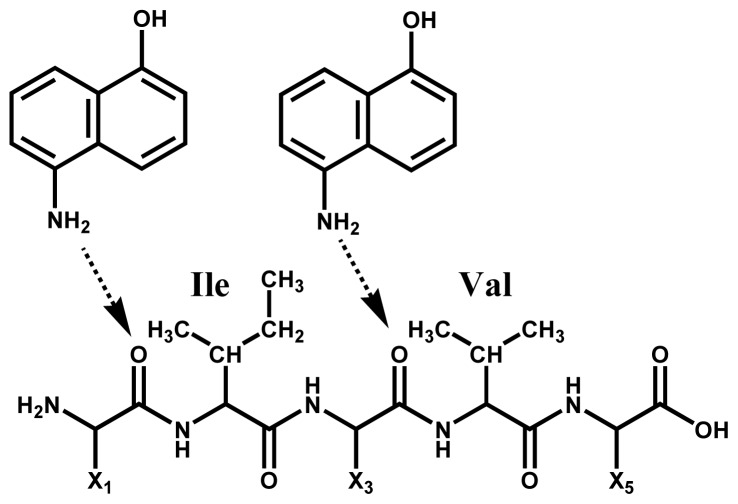
Steric hindrance by Ile and Val residues to form preliminary hydrogen-Bonding between matrix active-hydrogens and carbonyl oxygens of the backbone. The arrow represents hydrogen-bonding.

It is of importance to point out here that Ile and Val residues are more insensitive to ISD than the other amino acids. The peak abundance of c22 (Xxx-Val) and c24 (Xxx-Ile) in Figure 1 and c4 (Xxx-Ile) and c22–c24 (Xxx-Val23-Val24) in Figure 3 is very much lower than that of other peaks. This indicates that the *N*-terminal side carbonyl oxygen of the Ile and Val residues are prevented from forming interactions with matrix molecules. This inhibition may be due to a steric hindrance of bulky side chains of the Ile and Val residues (Scheme 4). It would be expected that such steric hindrance would result in low accessibility of hydrogen-radicals for the carbonyl oxygens of the backbone. This indicates that the access of hydrogen-radicals or matrix active-hydrogens to the backbone carbonyl oxygens of protein molecules is a principal factor in determining the peak abundance of ISD fragment ions.

### 2.4. Discussion

Although it is difficult to estimate the secondary structure and conformation of protein molecules embedded in/on MALDI matrix crystals, the high susceptibility of Asp, Asn, Cys and Gly residues and the low susceptibility of Ile and Val residues to ISD may be rationalized from the standpoint of the accessibility of the peptide backbone by hydrogen-radicals or matrix active-hydrogens. The high susceptibility of Asp, Asn, Cys and Gly residues is attributable to the high accessibility of the backbone by hydrogen-radicals or matrix active-hydrogens in flexible turn regions, while the low susceptibility of Ile and Val residues can be attributed to steric hindrance which prevents the backbone carbonyl oxygens from interactions with matrix molecules.

We can compare the flexible amino acid residues as judged by means of the hydrogen-radical accessibility as presented here with those estimated by other factors representing the flexibility of proteins, such as the B-factor, the turn preference factor, the protection factor and the fluorescence decay factor, as summarized in Table 2. It is of interest that Asp, Asn and Gly residues are identified as flexible by all the measures of flexibility despite the different principles used in the different measures. It is especially, noteworthy that Asp, Asn, Gly and Cys residues are common residues as identified by turn preference by X-ray data and hydrogen-radical accessibility by MALD-ISD data. This suggests that protein molecules embedded in/on matrix crystals partly maintain their secondary structures such as α-helix, and that the hydrogen-radical access to the backbone carbonyl oxygens could be adopted as a factor for measuring protein flexibility.

## 3. Materials and Methods

### 3.1. Chemicals

The matrix material 5-amino-1-naphthol (5,1-ANL) was purchased from Tokyo Chemical Industry (Tokyo, Japan). Acetonitrile was purchased from Wako Pure Chemicals (Osaka, Japan). Water used in all experiments was purified using a MilliQ water purification system from Millipore (Billerica, MA, USA). Bovine serum albumin (*M*r 66,430.3), equine apo-myoglobin (*M*r 16,951.4) and human thioredoxin with His-Tag (GSSHHHHHHSSGLVPRGSH) (*M*r 13,769.6) were purchased from Sigma-Aldrich (St. Louis, MO, USA). All reagents were used without further purification.

### 3.2. Mass Spectrometry

MALDI-TOF mass spectra were acquired on a time-of-flight mass spectrometer AXIMA-CFR (Shimadzu, Kyoto, Japan) equipped with a nitrogen laser (337 nm wavelength) operating at a pulse rate of 10 Hz. The pulse width of the laser was 4 ns. The laser spot size on the target substrate was ca. 100 μm in diameter. The ions generated by MALDI were accelerated using 20 kV with delayed extraction. The analyzer was operated in linear mode and the ions were detected using a secondary electron multiplier. A total of 500 shots were accumulated for each mass spectrum acquisition. The reproducibility of all ISD spectra were confirmed by the peak intensity patterns for several runs using the raster function installed on the AXIMA-CFR mass spectrometer.

### 3.3. Sample Preparation

Analyte for the MALDI-TOF MS experiments was dissolved in water at a concentration of 20 pmol/μL. The matrix material was dissolved in water/acetonitrile (3:7, *v*/*v*). The matrix and analyte solutions were prepared without any additives such as acetic acid. A sample solution was prepared by mixing a volume of 10 μL of analyte solution with a volume of 10 μL of matrix solution. A volume of 1.0 μL of the sample solution was deposited onto a stainless-steel MALDI plate and the solvents were removed by allowing evaporation in air at room temperature.

### 3.4. Ab Initio Calculations

The initial structures of the ISD fragments, *i.e.*, closed shell fragments c and z and open shell radical fragments c• and z• for model tri-peptide Me-Ala-X-Ala-Me (X = Ala, Gly, Asp), were made by a CS Chem3D Ultra (CambridgeSoft, Cambridge, MA, USA). The input files were minimized under semi-empirical MO (PM7) run through Winmoster interface using MOPAC2010. The output files were minimized under *ab initio* methods with the DFT UB3LYP level of theory and 6-31 + G (d) basis set. All *ab initio* calculations were performed with the Gaussian 09 suite of programs installed in an HP EliteBook 8460w (Nippon Hewlett-Packard, Tokyo, Japan) with an i7-2630 QM CPU Q720@1.60GHz (Intel, CA, USA). For every optimized structure vibrational analysis was performed and the heat of formation ΔH and Gibbs free energy ΔG were obtained.

## 4. Conclusions

Although it had been believed that mass spectrometry (MS) is in a disadvantageous position for obtaining information about flexibility or secondary and tertiary structures of protein molecules, here we present a factor for estimating the flexible amino acid residues in intact proteins by using a method of in-source decay (ISD) coupled with MALDI MS. The discontinuous intense peaks observed in MALDI-ISD spectra of BSA, myoglobin and thioredoxin originate from one-side preferential cleavage at the N-Cα bond of Xxx-Asp, Xxx-Asn, Xxx-Cys and Gly-Xxx residues lying in turn regions determined by X-ray crystallography. The flexible residues Asp, Asn, Cys and Gly estimated with MALDI-ISD were compared with the residues estimated from other measures of protein flexibility, such as the B-factor, turn preference, protection and fluorescence decay factors. The comparison shows that Asp, Asn and Gly residues were common to all the measures, and that Asp, Asn, Cys and Gly residues were identified by the ISD based method described here and by turn preference factor based on the X-ray crystallography. The results obtained suggest that protein molecules embedded in/on MALDI matrix crystals partly maintain the α-helix. Furthermore, the reasons that Asp, Asn, Cys and Gly residues are more susceptible and Ile and Val residues less susceptible to ISD can be rationalized in terms of the accessibility of matrix hydrogen-radicals to the backbone carbonyl oxygens. This implies that the hydrogen-radical accessibility in the MALDI-ISD experiments could be adopted as a factor for measuring protein flexibility.

## Supplementary Information



## Figures and Tables

**Table 1.** Discontinuous Intense Fragment Ions and Cleavage Sites observed in Positive Ion ISD Spectra of Intact Proteins BSA, Myoglobin and Thioredoxin.

Protein	Fragment Ion and Cleavage Residues
BSA	c12 (Xxx-Asp), c15 (Gly-Xxx), c33 (Xxx-Cys)
Myoglobin	z′15 (Xxx-Arg), c19 (Xxx-Asp), c35 (Gly-Xxx), c43 (Xxx-Asp), c59 (Xxx-Asp), c80 (Gly-Xxx)
Thioredoxin	c3 (Lys-Xxx), c19 (Gly-Xxx), c25 (Xxx-Asp), c31 (Xxx-Cys), c34 (Xxx-Cys), c61 (Xxx-Cys), c68 (Xxx-Cys), c72 (Xxx-Cys), w33 (Xxx-Cys), w37 (Xxx-Cys), w44 (Xxx-Cys), w71 (Xxx-Cys), w74 (Xxx-Cys)

**Table 2.** Amino Acid Residues estimated from the B-Factor, the Turn Preference Factor, the Protection Factor, the Fluorescence Factor and the Hydrogen-Radical Accessibility Factor that represent Flexibility of Protein.

Factor for Protein Flexibility	Flexible Amino Acid Residue
B-factor [10]	Asp, Asn, Gly, Pro, Lys, Glu, Gln, Ser
Turn preference [11]	Asp, Asn, Gly, Cys, Pro, Ser
Protection [13]	Asp, Asn, Gly, Lys, Thr, Ile, Met
Fluorescence decay [14]	Asp, Asn, Gly, Ser, Ala
Hydrogen-radical accessibility	Asp, Asn, Gly, Cys
