# Supplementary material for: Access of Hydrogen-Radicals to the Peptide-Backbone as a Measure for Estimating the Flexibility of Proteins Using Matrix-Assisted Laser Desorption/Ionization Mass Spectrometry
Source: Int J Mol Sci. 2014 May 13;15(5):8428–42. doi: 10.3390/ijms15058428 (PMC4057740; doi:10.3390/ijms15058428)
Supplement: Supplementary file 1 [file ijms-15-08428-s001.pdf]

Supplementary Information

Supplementary Information 1. PDB (protein data bank) sequence data of equine apo-myoglobin, bovine serum albumin (BSA) and human thioredoxin.

Myoglobin (PDB: 2FRF)

---

1 GLSDGEWQQV LNVWGKVEAD IAGHGQEVLI RLFTGHPETL EKFDKFKHLK
51 TEAEMKASED LKKHGTVVLT ALGGILKKKG HHEAELKPLA QSHATKHKIP
101 IKYLEFISDA IIHVLHSKHP GDFGADAQGA MTKALELFRN DIAAKYKELG
151 FQG

BSA (PDB: 4F5S)

---

1 DTHKSEIAHR FKDLGEEHFK GLVLIAFSQY LQQCPFDEHV KLVNELTEFA
51 KTCVADESHA GCEKSLHTLF GDELCKVASL RETYGDMADC CEKQEPERNE
101 CFLSHKDDSP DLPKLKPDPN TLCDEFKADE KKFWGKYLYE IARRHPYFYA
151 PELLYYANKY NGVFQECCQA EDKGACLLPK IETMREKVLT SSARQRLRCA
201 SIQKFGERAL KAWSVARLSQ KFPKAEFVEV TKLVTDLTKV HKECCHGDLL
251 ECADDRADLA KYICDNQDTI SSKLKECCDK PLLEKSHCIA EVEKDAIPEN
301 LPPLTADFAE DKDVCKNYQE AKDAFLGSFL YEYSRRHPEY AVSVLLRLAK
351 EYEATLEECC AKDDPHACYS TVFDKLKHLV DEPQNLIKQN CDQFEKLGEY
401 GFQNALIVRY TRKVPQVSTP TLVEVSRSLG KVGTRCCTKP ESERMPCTED
451 YLSLILNRLC VLHEKTPVSE KVTKCCTESL VNRRPCFSAL TPDETYVPKA
501 FDEKLFTFHA DICTLPDTEK QIKKQTALVE LLKHKPKATE EQLKTVMENF
551 VAFVDKCCAA DDKEACFAVE GPKLVVSTQT ALA

Thioredoxin (PDB: 1AUC)

---

1 MVKQIESKTA FQEALDAAGD KLVVVDFSAT WCGPCKMIKP FFHSLSEKYS
51 NVIFLEVDVD DCQDVASECE VKCMPTFQFF KKGQKVGEFS GANKEKLEAT
101 INELV

Supplementary Information 2. ISD (in-source decay) fragment ions (intensity) observed in the ISD spectra of bovine serum albumin (BSA) (Table S1), equine apo-myoglobin (Table S2), and human thioredoxin (Table S3).

Table S1. ISD (in-source decay) spectra of bovine serum albumin (BSA).

N-Term. Residue	c_n	y_n	z_n	*C*-Term. Residue
His9				Val575
Arg10	c10 (868)			Leu574
Phe11	c11 (1777)			Lys573
Lys12	c12 (1726)			Pro572
Asp13	c13 (1185)			Gly571
Leu14	c14 (1187)			Glu570
Gly15	c15 (3038)			Val569
Glu16	c16 (1010)			Ala568
Glu17	c17 (941)			Phe567
His18	c18 (835)			Cys566
Phe19	c19 (549)	y19 (204)		Ala565
Lys20	c20 (773)	y20 (230)		Glu564
Gly21	c21 (850)			Lys563
Leu22	c22 (343)		z22 (172)	Asp562
Val23	c23 (675)			Asp561
Leu24	c24 (457)			Ala560
Ile25	c25 (520)			Ala559
Ala26	c26 (582)			Cys558
Phe27	c27 (658)			Cys557
Ser28	c28 (596)			Lys556
Gln29	c29 (355)			Asp555
Tyr30	c30 (309)			Val554
Leu31	c31 (377)			Phe553
Gln32	c32 (351)			Ala552
Gln33	c33 (4516)			Val551
Cys34				Phe550
Pro35	c35 (131)			Asn549
Phe36	c36 (141)			Glu548
Asp37	c37 (88)		z37 (1337)	Met547
Glu38	c38 (55)			Val546
His39	c39 (49)			Thr545
Val40	c40 (47)			Lys544
Lys41	c41 (35)			Leu543
Leu42				Gln542
Val43	c43 (71)			Glu541
Asn44	c44 (36)			Glu540
Glu45	c45 (31)			Thr539
Leu46				Ala538
Thr47				Lys537
Glu48				Pro536
Phe49				Lys535

Table S2. ISD spectra of myoglobin.

N-Term. Residue	c_n	y_n	z_n	*C*-Term. Residue
Ala50				His534
Gln9		y9 (26)	z9 (18)	Lys145
Val10	c10 (22)	y10 (20)	z10 (34)	Ala144
Leu11	c11 (154)	y11 (42)	z11 (30)	Ala143
Asn12	c12 (30)		z12 (28)	Ile142
Val13	c13 (30)		z13 (165)	Asp141
Trp14	c14 (48)		z14 (303)	Asn140
Gly15	c15 (65)		z15 (1583)	Arg139
Lys16	c16 (119)		z16 (580)	Phe138
Val17	c17 (355)		z17 (661)	Leu137
Glu18	c18 (339)	y18 (131)	z18 (667)	Glu136
Ala19	c19 (1291)	y19 (96)	z19 (382)	Leu135
Asp20	c20 (144)	y20 (173)	z20 (423)	Ala134
Ile21	c21 (193)	y21 (567)	z21 (644)	Lys133
Ala22	c22 (567)	y22 (155)	z22 (409)	Thr132
Gly23	c23 (468)	y23 (137)	z23 (812)	Met131
His24	c24 (753)	y24 (266)	z24 (908)	Ala130
Gly25	c25 (777)	y25 (255)	z25 (1091)	Gly129
Gln26	c26 (552)	y26 (180)	z26 (506)	Gln128
Glu27	c27 (311)	y27 (246)	z27 (609)	Ala127
Val28	c28 (245)	y28 (442)	z28 (1097)	Asp126
Leu29	c29 (152)	y29 (207)	z29 (421)	Ala125
Ile30	c30 (358)	y30 (182)	z30 (348)	Gly124
Arg31	c31 (1073)	y31 (320)	z31 (492)	Phe123
Leu32	c32 (964)	y32 (192)	z32 (746)	Asp122
Phe33	c33 (577)			Gly121
Thr34	c34 (509)	y34 (242)		Pro120
Gly35	c35 (2383)		z35 (365)	His119
His36		y36 (79)	z36 (225)	Lys118
Pro37	c37 (544)	y37 (109)	z37 (182)	Ser117
Glu38	c38 (612)		z38 (167)	His116
Thr39	c39 (600)		z39 (84)	Leu115
Leu40	c40 (444)		z40 (88)	Val114
Glu41	c41 (512)		z41 (132)	His113
Lys42	c42 (560)		z42 (70)	Ile112
Phe43	c43 (1316)		z43 (69)	Ile111
Asp44	c44 (431)		z44 (136)	Ala110
Lys45	c45 (386)	y45 (78)	z45 (214)	Asp109
Phe46	c46 (350)			Ser108
Lys47	c47 (294)		z47 (79)	Ile107
His48	c48 (325)		z48 (138)	Phe106
Leu49	c49 (201)		z49 (107)	Glu105
Lys50	c50 (142)			Leu104
Thr51	c51 (226)		z51 (94)	Tyr103
Glu52	c52 (165)		z52 (69)	Lys102

Table S2. *Cont.*

N-Term. Residue	c_n	y_n	z_n	*C*-Term. Residue
Ala53	c53 (127)		z53 (39)	Ile101
Glu54	c54 (194)			Pro100
Met55	c55 (93)		z55 (53)	Ile99
Lys56	c56 (94)		z56 (34)	Lys98
Ala57	c57 (180)	y57 (34)	z57 (25)	His97
Ser58	c58 (187)			Lys96
Glu59	c59 (197)		z59 (29)	Thr95
Asp60	c60 (161)		z60 (43)	Ala94
Leu61	c61 (44)		z61 (52)	His93
Lys62	c62 (79)		z62 (42)	Ser92
Lys63	c63 (71)		z63 (33)	Gln91
His64	c64 (48)		z64 (23)	Ala90
Gly65	c65 (93)		z65 (29)	Leu89
Thr66	c66 (22)			Pro88
Val67	c67 (31)		z67 (33)	Lys87
Val68	c68 (22)		z68 (18)	Leu86
Leu69	c69 (23)		z69 (10)	Glu85
Thr70	c70 (66)		z70 (11)	Ala84
Ala71	c71 (20)		z71 (13)	Glu83
Leu72	c72 (23)		z72 (16)	His82
Gly73	c73 (65)			His81
Gly74	c74 (30)			Gly80
Ile75	c75 (30)			Lys79
Leu76	c76 (17)			Lys78
Lys77	c77 (38)		z77 (36)	Lys77
Lys78	c78 (13)			Leu76
Lys79	c79 (15)			Ile75
Gly80	c80 (45)			Gly74
His81	c81 (15)			Gly73
His82	c82 (11)			Leu72
Glu83	c83 (12)			Ala71
Ala84	c84 (7)			Thr70
Glu85	c85 (6)			Leu69
Leu86	c86 (11)			Val68

Table S3. ISD spectra of thioredoxin.

N-Term. Residue	c_n	y_n	z_n	w_n	*C*-Term. Residue
Met1	c1 (1035)	y20 (105)	z20 (724)		Val86
Val2	c2 (1673)		z21 (458)		Lys85
Lys3	c3 (2251)		z22 (702)		Gln84
Gln4	c4 (1009)	y23 (474)	z23 (254)		Gly83
Ile5	c5 (1681)	y24 (571)	z24 (591)		Lys82
Glu6	c6 (2285)	y25 (317)	z25 (523)		Lys81
Ser7	c7 (2030)	y26 (870)	z26 (2030)		Phe80

Table S3. *Cont.*

N-Term. Residue	c_n	y_n	z_n	w_n	*C*-Term. Residue
Lys8	c8 (1948)				Phe79
Thr9	c9 (1914)	y28 (119)	z28 (894)		Gln78
Ala10	c10 (1591)		z29 (1542)		Phe77
Phe11	c11 (1312)	y30 (57)	z30 (234)		Thr76
Gln12	c12 (1373)	y31 (357)			Pro75
Glu13	c13 (1194)	y32 (1164)	z32 (1593)		Met74
Ala14	c14 (1307)	y33 (226)		w33 (4941)	Cys73
Leu15	c15 (1740)		z34 (366)		Lys72
Asp16	c16 (1265)		z35 (474)		Val71
Ala17	c17 (828)				Glu70
Ala18	c18 (824)			w37 (3230)	Cys69
Gly19	c19 (2308)		z38 (255)		Glu68
Asp20	c20 (1113)	y39 (48)	z39 (97)		Ser67
Lys21	c21 (993)	y40 (43)	z40 (177)		Ala66
Leu22	c22 (221)	y41 (51)	z41 (61)		Val65
Val23	c23 (189)	y42 (80)	z42 (173)		Asp64
Val24	c24 (325)	y43 (193)	z43 (150)		Gln63
Val25	c25 (1245)	y44 (89)		w44 (1520)	Cys62
Asp26	c26 (1075)		z45 (353)		Asp61
Phe27	c27 (628)		z46 (119)		Asp60
Ser28	c28 (585)		z47 (75)		Val59
Ala29	c29 (479)	y48 (35)	z48 (83)		Asp58
Thr30	c30 (391)	y49 (605)			Val57
Trp31	c31 (2428)				Glu56
Cys32	c32 (645)				Leu55
Gly33			z52 (44)		Phe54
Pro34	c34 (1397)				Ile53
Cys35	c35 (127)				Val52
Lys36	c36 (118)				Asn51
Met37	c37 (52)		z56 (23)		Ser50
Ile38	c38 (215)		z57 (37)		Tyr49
Lys39					Lys48
Pro40	c40 (134)		z59 (62)		Glu47
Phe41	c41 (251)				Ser46
Phe42	c42 (199)		z61 (104)		Leu45
His43	c43 (236)		z62 (128)		Ser44
Ser44	c44 (228)		z63 (95)		His43
Leu45	c45 (195)				Phe42
Ser46	c46 (264)		z65 (25)		Phe41
Glu47	c47 (215)				Pro40
Lys48	c48 (64)				Lys39
Tyr49	c49 (63)				Ile38
Ser50	c50 (122)				Met37
Asn51	c51 (20)				Lys36
Val52	c52 (10)			w71 (82)	Cys35

Table S3. *Cont.*

N-Term. Residue	c_n	y_n	z_n	w_n	*C*-Term. Residue
Ile53	c53 (15)				Pro34
Phe54	c54 (21)				Gly33
Leu55	c55 (26)			w74 (51)	Cys32
Glu56	c56 (17)				Trp31
Val57	c57 (38)				Thr30
Asp58	c58 (13)				Ala29
Val59	c59 (25)				Ser28
Asp60	c60 (32)				Phe27
Asp61	c61 (155)				Asp26
Cys62	c62 (16)				Val25
Gln63	c63 (24)				Val24
Asp64					Val23
Val65					Leu22
Ala66					Lys21
Ser67					Asp20
Glu68	c68 (41)				Gly19
Cys69					Ala18
Glu70					Ala17
Val71					Asp16
Lys72	c72 (25)				Leu15
Cys73	c73 (11)				Ala14
Met74					Glu13
Pro75					Gln12
Thr76					Phe11
Phe77					Ala10
Gln78					Thr9
Phe79					Lys8
Phe80					Ser7
Lys81					Glu6
Lys82					Ile5
Gly83					Gln4
Gln84					Lys3
Lys85					Val2
Val86					Met1

© 2014 by the authors; licensee MDPI, Basel, Switzerland. This article is an open access article distributed under the terms and conditions of the Creative Commons Attribution license (http://creativecommons.org/licenses/by/3.0/).
